# Clinical value of the renal pathologic scoring system in complement-mediated thrombotic microangiopathy

**DOI:** 10.1080/0886022X.2022.2161396

**Published:** 2023-01-17

**Authors:** Fei-Fei Chen, Xiao-Juan Yu, Hui Wang, Xu Zhang, Ying Tan, Zhen Qu, Su-Xia Wang, Feng Yu, Min Chen, Ming-Hui Zhao

**Affiliations:** a Renal Division, Department of Medicine, Peking University First Hospital; Institute of Nephrology, Peking University; Renal Pathology Center, Institute of Nephrology, Peking University First Hospital; Key Laboratory of Renal Disease, Ministry of Health of China; Key Laboratory of CKD Prevention and Treatment, Ministry of Education of China, Beijing, PR China; b Department of Electron Microscopy, Pathological Centre, Peking University First Hospital, Beijing, PR China; c Department of Nephrology, Peking University International Hospital, Beijing, PR China; d Peking-Tsinghua Center for Life Sciences, PR China

**Keywords:** TMA, C-TMA, microangiopathy, kidney, pathology

## Abstract

**Objectives:**

This study was initiated to establish a renal thrombotic microangiopathy (TMA) scoring system based on clinical needs and investigate its predictive value for patients’ long-term outcomes.

**Methods:**

Kidney biopsy-proven Complement-mediated TMA (C-TMA) patients from January 2000 to December 2017 in Peking University First Hospital were retrospectively studied. Both acute and chronic TMA-related lesions, including 15 pathologic indices, were semiquantitatively scored. The interobserver and intraobserver reproducibility and correlation between the pathologic indices and clinical parameters were analyzed. Furthermore, the patients were divided into 2 groups by dialysis use at baseline, and the association of these pathologic indices with their prognostic outcomes was assessed between the two groups.

**Results:**

Ninety-two patients with renal biopsy-proven C-TMA were enrolled. All fifteen included pathology indices showed good or moderate interobserver and intraobserver reproducibility and correlated well with several clinical parameters. Several clinicopathological indices were worse in the dialysis group than in the nondialysis group, such as serum creatinine, hemoglobin, platelet count, and estimated glomerular filtration rate. Moreover, morphologic features in the dialysis group presented with more severe vascular lesions. Interstitial fibrosis and chronic tubulointerstitial lesions were related to a trend of high risk of continuous dialysis in the dialysis group. Based on univariate and multivariable Cox regression analysis, more severe glomerular lesions, including glomerular mesangiolysis, glomerular basement membrane double contours and glomerular mesangial proliferation, were identified as risk factors predicting worse prognosis.

**Conclusions:**

Our renal C-TMA semiquantitative scoring system is reliable with good reproducibility and prognostic value in clinical practice, which needs further validation.

## Introduction

Thrombotic microangiopathy (TMA) is characterized by various causes that induce microvascular endothelial injury followed by subsequent pathologic manifestations, including subendothelial edema and microthrombi formation, and clinically manifests as consumptive thrombocytopenia, microangiopathic hemolysis, and multiple organ ischemic damage [1]. With the increased understanding of disease mechanisms including the impact of genetic background and triggers, the concept of the ‘complement-mediated TMA (C-TMA)’ has emerged [2,3]. C-TMA is a rare genetic or acquired life-threatening disease of uncontrolled complement activation. Recently, studies of complement genetics have transformed the landscape of TMA [4–7], and complement genetics also reclassified pregnancy- and postpartum-associated hemolytic uremic syndrome (HUS) as the spectrum of complement-mediated thrombotic microangiopathy, resulting in improved management of women with this severe disorder [4,5,8,9].

The kidney is one of the most commonly involved organs with the most easily obtained biopsied samples, which makes it a feasible way to explore TMA pathologic changes, although associated studies are rare. The typical pathologic features of TMA include microvascular endothelial injury presented as subendothelial myxoid edema and secondary microthrombi as acute lesions in both glomeruli and arterioles/arteries, while the double-contours of GBM and onion skin lesions of arterioles/arteries are chronic lesions [10–15]. Interestingly, some TMA patients have pathological evidence of microvascular endothelial injury without microthrombi formation. These patients usually do not present with systemic manifestations, such as microangiopathic hemolytic anemia and thrombocytopenia. Thus, a recent Kidney Disease Improving Global Outcomes (KDIGO) conference [16] suggested that microangiopathy, a new terminology to describe the generous TMA patients, should be concerned although it needs further specification of whether thrombosis was present. However, there is still a lack of standard consensus of the renal TMA pathological scoring system, and the clinical significance of different morphologic features of the disease, especially its correlation with renal outcomes, remains to be further elucidated.

Herein, we aimed to identify different renal pathological features in TMA patients and propose a scoring system based on a well-defined Chinese cohort.

## Materials and methods

### Patients

**Figure 1.**
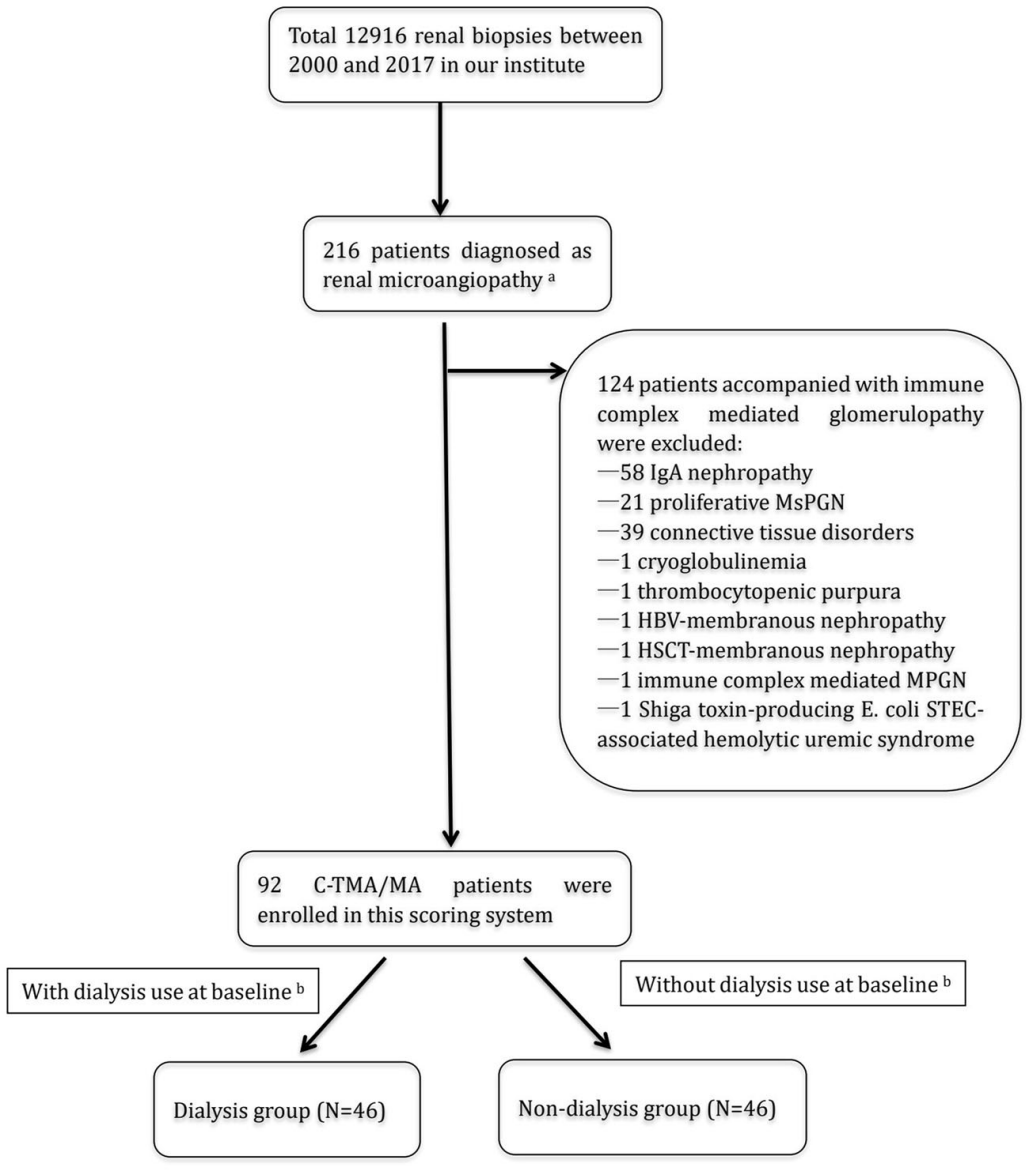
Patient recruitment chart in the C-TMA cohort. ^a^Patients fulfilling at least one of the following pathological criteria were diagnosed as renal TMA: (1) artery/arteriole thrombi formation; (2) glomerular thrombi formation; (3) Onion skin change of artery/arteriole; (4) Myxoid change of artery/arteriole; (5) glomerular subendothelial edema on electron microscopy. ^b^Patients were grouped by dialysis use at baseline. Abbreviations: MsPGN: Mesangial proliferative glomerulonephritis; MPGN: Membranoproliferative glomerulonephritis.

Kidney biopsy-proven TMA patients from January 2000 to December 2017 at Peking University First Hospital were enrolled as shown in Figure 1. Patients fulfilling at least one of the following pathological criteria were diagnosed with renal TMA [7,10,12–15]: (1) arteriolar/arterial thrombi formation; (2) glomerular thrombi formation; (3) onion skin change of arterioles/arteries; (4) myxoid edema of arterioles/arteries; and (5) glomerular subendothelial edema on electron microscopy. C-TMA was diagnosed after exclusion of the following conditions [4,7,14,16]: (1) thrombotic thrombocytopenic purpura (TTP); (2) Shiga toxin-producing E. coli (STEC)-associated HUS; (3) coexisting immune complex-mediated diseases; (4) secondary TMA, including drugs, malignant diseases, and autoimmune diseases. Renal biopsies with <10 scorable glomeruli or <6 scorable arterioles/arteries were also excluded.

The research was in compliance with the Declaration of Helsinki, and the design of this work was specifically approved by the ethical committees of Peking University First Hospital (approval number: 2014 [739]).

### Clinical and laboratory assessment

The clinical data were extracted from the electronic medical records of Peking University First Hospital, including age, sex, serum creatinine, proteinuria, serum albumin (ALB), hemoglobin, platelet count, lactate dehydrogenase (LDH) level, serum C3, serum C4, hematuria, and extrarenal organ involvement. The serum creatinine level, age, and sex were used for the GFR estimates following the CKD-EPI estimated glomerular filtration rate (eGFR) equation [17]. Clinical parameters were measured within 3 months of the date of biopsy.

Therapeutic regimens included plasma therapy (plasmapheresis or plasma infusion), immunosuppressants (including glucocorticoids), and renin–angiotensin–aldosterone system (RAAS) inhibitors. No patient in our cohort received eculizumab due to inaccessibility in China.

As end-stage kidney disease (ESKD) occurred at the onset of some patients in our cohort, the enrolled patients were grouped by dialysis use at baseline. The endpoints during follow-up were defined as in a previous report [8,15,18,19]. For the dialysis group, the primary endpoint was discontinuation of dialysis. For the nondialysis group, the composite endpoints were defined by all-cause death or ESKD.

## Renal histopathology

### Renal pathological evaluation

Renal biopsy specimens were examined by direct immunofluorescence, light microscopy (LM), and electron microscopy and were fixed in 4% buffered formaldehyde for LM. Consecutive serial 3 µm sections were used for histological staining, including hematoxylin and eosin, periodic acid-silver methenamine, periodic acid-Schiff staining, and Masson’s trichrome. Immunofluorescence staining of the kidney was performed on fixed-frozen sections, including IgA, IgM, IgG, C1q, C3, albumin, and fibrinogen. A semiquantitative approach was used to score the immunofluorescence labeling (0–3).

Two nephropathologists who were blinded to the patients’ data and scores of the other observer evaluated the biopsies separately. Differences in scoring between the pathologists were resolved by rereviewing the biopsies and thus reaching a consensus. Semiquantitative scoring was based on light microscopy, except for subendothelial edema on electron microscopy.

### Semiquantitative evaluation of renal microangiopathy changes

Glomerular and arteriolar parameters were analyzed as semiquantitative variables and expressed as the percentage involvement of all scoreable glomeruli and arterioles based on Banff type [20].

The scoring criteria were composed of the following three parts containing 15 indices (details are shown in Supplementary Table S1).

**Table 1.** General data of patients with aHUS, P-HUS, and malignant hypertension of unknown cause.

Clinical evaluation		Laboratory Assessment		Medications, no. (%)	
Sex (male/female), no.	59/33	Serum creatinine (mg/dL), median (range)	5.83 (0.62–33.03)	PE and/or PI	26 (28.3)
Age (years), mean ± SD	36.64 ± 9.55			Steroids and other immunosuppressants	33 (35.9)
Fever, no. (%)	8 (8.7)	Urine protein (g/24h), median (range)	2.11 (0.03–14.31)		
Diabetes, no. (%)	3 (3.3)			Cyclophosphamide	10 (10.9)
Hematuria, no. (%)	40 (43.5)	Albumin (g/L), mean ± SD	37.05 ± 5.99	RAAS blockades	68 (73.9)
Liver involvement, no. (%)	8 (8.7)	Hemoglobin (g/dL), median (range)	91 (33–170)	Follow-up	
Cardiac involvement, no. (%)	1 (1.1)			Duration of follow-up, month, median (range)	35 (1–209)
Neurologic disorder, no. (%)	8 (8.7)	Platelet count (×10^3^/mL), median (range)	149 (15–397)		
Renal cortical necrosis, no. (%)	6 (6.5)			Dialysis at baseline, no. (%)	46 (50.0)
Hematologic involvement, no. (%)	43 (46.7)	eGFR (mL/min/1.73m^2^), median (range)	10.10 (1.50–115.10)	Patients on dialysis at baseline who discontinued during the study period, no. (%)^a^	26 (28.3)
Musculoskeletal system involvement, no. (%)	1 (1.1)				
Etiology, no. (%)		LDH (U/L), median (range), *n* = 69	367 (103–7226)	Nondialysis at baseline, no. (%)	46 (56.5)
aHUS	19 (20.7)	Serum C3 (g/L), mean ± SD, *n* = 85	0.92 ± 0.26	Composite events, no. (%)^b^	19 (41.3)
P-HUS	20 (21.7)	Serum C4 (g/L), mean ± SD, *n* = 72	0.25 ± 0.10	Death, no. (%)^b^	1 (5.3)
Malignant hypertension of unknown cause	53 (57.6)			ESKD, no. (%)^b^	19 (41.3)

Abbreviations: TMA: Thrombotic microangiopathy; eGFR: Estimated glomerular filtration rate; LDH: Lactate dehydrogenase; PE: Plasma exchange; PI: Plasma infusion; RAAS: Rennin–angiotensin–aldosterone system (include angiotensin-converting enzyme inhibitor and angiotensin receptor blocker); ESKD: End-stage kidney disease; aHUS: Atypical hemolytic uremic syndrome; P-HUS: Pregnancy- and postpartum-associated hemolytic uremic syndrome.

Normally distributed variables were expressed as mean ± SD. Nonparametric variables were expressed as median and categorical variables were expressed in percentages.

^a^ Among 46 patients with dialysis use at baseline, 26 patients discontinue dialysis at the end of study.

^b^ Among 46 patients without dialysis use at baseline, 19 patients had composite events, 1 patient succumbed to multiple organ failure, and 19 patients developed ESKD at the end of study.

Part 1: Glomerular microangiopathic lesions, including acute glomerular microangiopathic lesions (glomerular endothelial cell swelling and proliferation, subendothelial edema on electron microscopy, glomerular mesangiolysis, and glomerular necrosis/thrombi) and chronic glomerular microangiopathic lesions (glomerular basement membrane double contours, glomerular mesangial proliferation, ischemic wrinkle of glomerular basement membrane double, and global and segmental glomerulosclerosis). The details are shown in Supplementary Table S1 (A).

Part 2: Vascular microangiopathic lesions, including acute vascular microangiopathic lesions (vascular endothelial cell swelling and subintimal myxoid edema and arteriolar/arterial fibrinoid necrosis/thrombi) and chronic vascular microangiopathic lesions (onion skin lesion, intimal fibrosis, and arteriolar hyalinosis). The details are shown in Supplementary Table S1 (B).

Part 3: Chronic tubulointerstitial lesions, including tubular atrophy and interstitial fibrosis. The details are shown in Supplementary Table S1 (C).

### Genetic testing

Genomic DNA was extracted from peripheral blood cells of patients. An ultraviolet spectrophotometer was used to detect the RNA concentration, and agarose gel electrophoresis was used to measure the RNA quality. Whole-exome sequencing (WES) was completed by the Huada Gene Institute (Beijing, China).

### Statistical analysis

SPSS software (version 24; SPSS Inc., Chicago, IL, USA) was used for statistical analysis. Normally distributed variables are expressed as the means ± standard deviation (SD), and differences in quantitative parameters among groups are assessed with Student’s *t*-test or one-way ANOVA for two or more independent samples as appropriate. Nonparametric variables are expressed as the medians with range (minimum–maximum) and compared using the Mann–Whitney *U* test or Kruskal–Wallis *H* test. Categorical variables are expressed as the percentages. The Kruskal–Wallis *H* test was used to compare quantitative versus categorical variables, and the chi-square test was used to compare categorical versus categorial variables. Reproducibility was assessed for each variable of the extended pathology dataset using intraclass correlation coefficients (ICCs) [21]. All of these measures were used to assess the agreement of the scoring by two pathologists. The ICC is a measure of reproducibility applicable to multiple raters. By convention, a value of >0.80 is good or very good reproducibility, 0.40–0.80 is moderate, and <0.40 is poor or fair [22,23].

The Kaplan–Meier curves were generated to analyze the risk factor for prognosis and compared by the log-rank test. The correlation analysis between pathological features and clinical data was based on the semiquantitative degree of the pathological lesions using Spearman’s test. The Cox proportional hazards regression model was used to quantify the relationship between exposures and the outcomes (outcomes in dialysis group: discontinuing dialysis; outcomes in nondialysis group: all-cause death or ESKD). The results were expressed as the hazard ratios (HRs) with 95% CIs. *p* < 0.05 was considered to be statistically significant. The factors that were verified by univariate Cox regression analysis were entered into the multivariate Cox regression analysis based on the rationale of stepwise multivariable Cox regression analysis, which considered the pathology variables in addition to the initial age, sex, and associated disorders. Model ^a^ was adjusted for traditional clinical markers (age, sex, and eGFR) [24], and Model^b^ was adjusted for variables based on the univariate analysis (age, sex, eGFR, proteinuria, and hematuria). Differences were considered significant when *p* < 0.05.

## Results

### Patient baseline data

The study included 92 Chinese renal TMA patients (Figure 1). Their baseline data are summarized in Table 1. The cohort included 59 male (64.1%) and 33 female (35.9%) patients, and the mean age was 34.64 ± 9.55 years. Among them, 29 patients (29/92, 31.5%) underwent genetic testing and complement genes variants were found in 17 patients (17/29, 58.6%), including complement factor B, complement factor P, complement factor H, Complement Factor H Related Protein 1, C3, C5, C7, C8, C9, plasminogen, and thrombomodulin.

The median follow-up time was 35 months (range, 1–209 months). The enrolled patients were grouped based on whether they were on dialysis or not at baseline. In the dialysis group, 26 patients (26/46, 56.5%) discontinued dialysis after a median of 2 months (range: 1–13 months). In the nondialysis group, one patient (1/46, 2.2%) died due to multiple organ failure, and 19 patients (19/46, 41.3%) developed ESKD with a median of 35 months (range, 3–209 months).

### Histological lesion scoring

**Figure 2.**
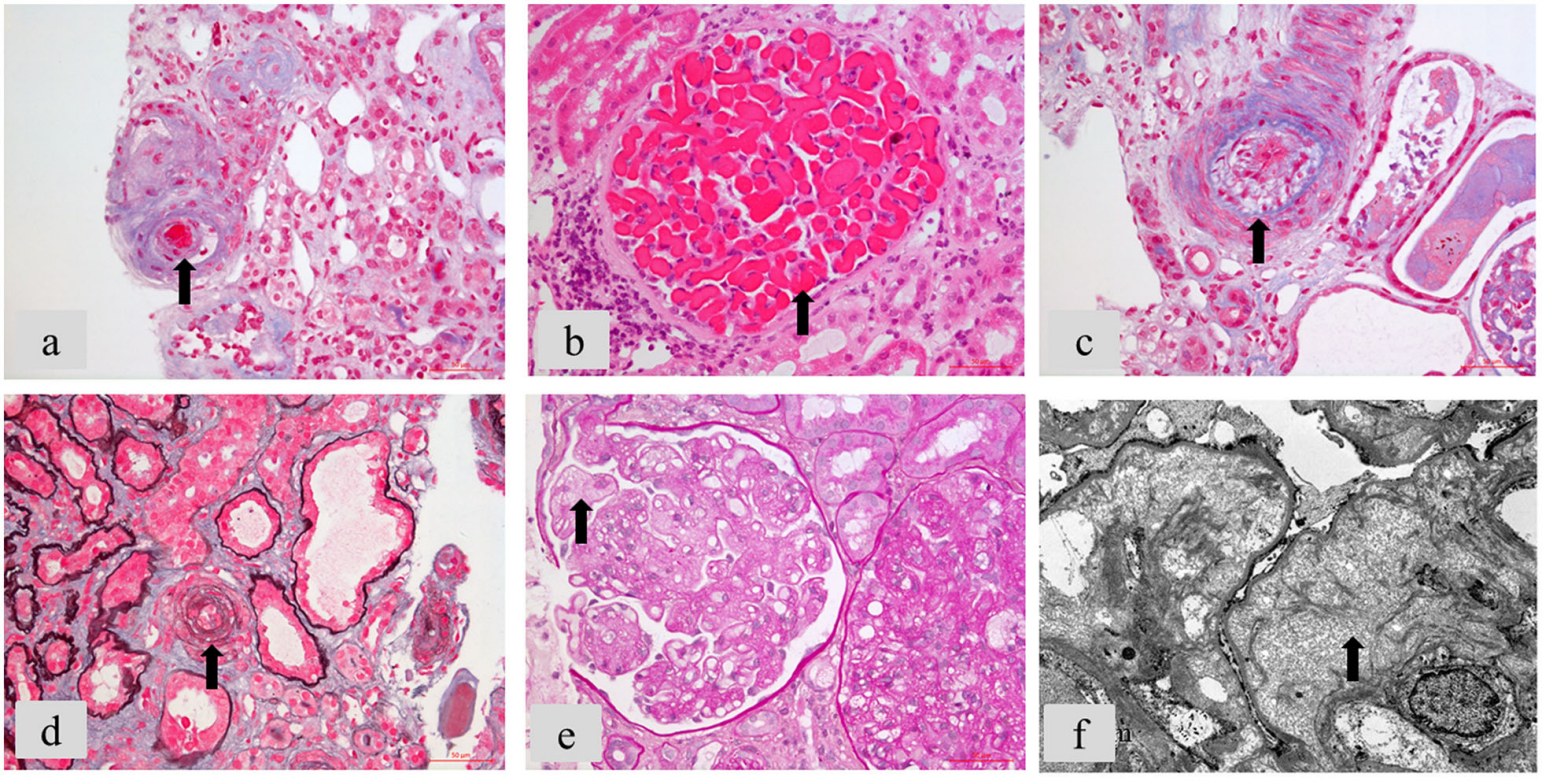
The classic renal pathological lesions of C-TMA. Arrows pointed to typical changes. (a) Arteriole thrombi (Masson’s trichrome staining, ×400). (b) Glomerular thrombi (Hematoxylin and eosin (H&E) staining, ×400). (c) Subintimal myxoid edema of artery (Masson’s trichrome staining, ×400). (d) Onion skin lesion of arteriole (Periodic Acid-Silver Methenamine and Masson’s trichrome staining). (e) Glomerular endothelial swelling, proliferation, and double contour formation (Periodic acid-Schiff staining, ×400). (f) Subendothelial edema of glomeruli by electron microscopy (×8000).

The typical renal pathological TMA lesions are presented in Figure 2, and their distributions in our cohort are shown in Supplementary Table S2.

**Table 2.** Pathology dataset: inter-observer reproducibility.

Parameters	ICC
Glomerular endothelial cell swelling and proliferation	0.847***
Glomerular subendothelial edema on electron microscopy	0.876***
Glomerular mesangiolysis	0.873***
Glomerular necrosis/thrombi	1.000***
GBM double contours	0.958***
Glomerular mesangial proliferation	0.659***
Ischemic wrinkle of GBM	0.945***
Global and segmental glomerulosclerosis	0.868***
Vascular endothelial cell swelling and subintimal myxoid edema	0.945***
Arteriolar/artery fibrinoid necrosis/thrombi	1.000***
Vascular onion skin lesion	0.943***
Intimal fibrosis of artery	0.954***
Arteriolar hyalinosis	0.923***
Tubular atrophy	0.882***
Interstitial fibrosis	0.910***

*** *p* < 0.001.

Abbreviations: GBM: Glomerular basement membrane; ICC: Intraclass correlation coefficient.

### Extended pathology dataset and reproducibility of histological lesions

A score sheet was completed by individual pathologists and the reproducibility of the agreement of the scoring by the two pathologists was further assessed. Overall, 15 histopathologic variables were assessed, and reproducibility was assessed statistically using intraclass correlation coefficients (ICCs), which are summarized in Table 2.

Based on the ICC scores, these lesions were divided into 3 groups as follows:

Group 1: Indices with values of good or very good reproducibility (ICC > 0.80) included glomerular endothelial cell swelling and proliferation, subendothelial edema on electron microscopy, glomerular mesangiolysis, glomerular necrosis/thrombi, glomerular basement membrane (GBM) double contours, ischemic wrinkle of GBM, global and segmental glomerulosclerosis, vascular endothelial cell swelling and subintimal myxoid edema, arteriolar/arterial fibrinoid necrosis/thrombi, onion skin lesion, intimal fibrosis, arteriolar hyalinosis, tubular atrophy, and interstitial fibrosis.

Group 2: Indices with moderate reproducibility (0.40 ≤ ICC ≤ 0.80), only glomerular mesangial proliferation.

Group 3: Indices with poor or fair reproducibility (ICC < 0.40), none.

Then, intraobserver reproducibility was further performed, which also showed moderate or good reproducibility of all abovementioned histopathologic variables. More details are listed in Supplementary Table S3.

**Table 3.** Associations between pathological indices and clinical features at the time of renal biopsy.

	Proteinuria		Serum albumin		Serum creatinine	
	*r*	*p*	*r*	*p*	*r*	*p*
Glomerular endothelial cell swelling and proliferation (0–3)	0.165	0.115	−0.313	0.002**	0.104	0.325
Glomerular subendothelial edema on electron microscopy (0–3)	0.017	0.874	−0.151	0.150	0.261	0.012*
Glomerular mesangialysis (0–1)	0.104	0.323	−0.103	0.326	−0.064	0.544
Glomerular thrombi/necrosis (0–2)	0.279	0.007**	−0.16	0.127	0.193	0.066
Acute glomerular microangiopathic lesions (0–9)	0.119	0.260	−0.374	<0.001***	0.363	<0.001***
Glomerular basement membrane double contour (0–3)	0.335	0.001**	−0.312	0.002**	0.095	0.367
Mesangial proliferation (0–3)	0.269	0.010*	−0.225	0.031*	0.102	0.332
Ischemic wrinkle of glomeruli (0–3)	−0.248	0.017*	0.003	0.997	0.375	<0.001***
Global and segmental sclerosis (0–3)	0.148	0.158	0.091	0.388	−0.094	0.375
Chronic glomerular microangiopathic lesions (0–12)	0.088	0.404	−0.142	0.176	0.343	<0.001***
Endothelial swelling and subintimal myxoid edema of artery/arteriole (0–3)	−0.063	0.550	−0.008	0.942	0.305	0.003**
Arteriolar/artery necrosis/thrombi (0–2)	0.119	0.260	−0.196	0.061	0.306	0.003**
Acute vascular microangiopathic lesions (0–5)	0.017	0.871	−0.093	0.377	0.352	0.001**
Arteriolar onion skin lesions (0–3)	−0.226	0.030*	0.082	0.438	0.324	0.002**
Intimal fibrosis of artery (0–3)	−0.071	0.504	0.171	0.103	−0.21	0.044*
Arteriolar hyalinosis (0–3)	−0.075	0.480	0.044	0.675	−0.178	0.089
Chronic vascular microangiopathic lesions (0–9)	−0.203	0.052	0.177	0.092	−0.013	0.901
Tubular atrophy (0–3)	0.163	0.120	−0.06	0.569	0.241	0.021*
Interstitial fibrosis (0–3)	0.159	0.129	−0.06	0.573	0.18	0.087
Chronic tubulointerstitial lesions (0–6)	0.158	0.133	−0.062	0.558	0.219	0.036*

	eGFR		Hemoglobin		Platelet count	
	*r*	*p*	*r*	*p*	*r*	*p*
Glomerular endothelial cell swelling and proliferation (0–3)	−0.128	0.225	−0.352	0.001**	−0.227	0.029*
Glomerular subendothelial edema on electron microscopy (0–3)	−0.254	0.015*	−0.154	0.142	−0.138	0.191
Glomerular mesangialysis (0–1)	0.069	0.511	−0.073	0.492	−0.148	0.159
Glomerular thrombi/necrosis (0–2)	−0.214	0.041*	−0.312	0.002**	−0.087	0.407
Acute glomerular microangiopathic lesions (0–9)	−0.383	<0.001***	−0.425	<0.001***	−0.330	0.001**
Glomerular basement membrane double contour (0–3)	−0.126	0.231	−0.324	0.002**	−0.157	0.135
Mesangial proliferation (0–3)	−0.078	0.460	−0.185	0.077	0.039	0.711
Ischemic wrinkle of glomeruli (0–3)	−0.357	<0.001***	−0.231	0.027*	−0.161	0.126
Global and segmental sclerosis (0–3)	0.129	0.219	0.295	0.004**	0.347	0.001**
Chronic glomerular microangiopathic lesions (0–12)	−0.298	0.004**	−0.193	0.065	−0.004	0.968
Endothelial swelling and subintimal myxoid edema of artery/arteriole (0–3)	−0.304	0.003**	−0.200	0.056	−0.309	0.003**
Arteriolar/artery necrosis/thrombi (0–2)	−0.302	0.003**	−0.325	0.002**	−0.359	<0.001***
Acute vascular microangiopathic lesions (0–5)	−0.350	0.001**	−0.275	0.008**	−0.377	<0.001***
Arteriolar onion skin lesions (0–3)	−0.224	0.032*	0.096	0.365	0.112	0.290
Intimal fibrosis of artery (0–3)	0.240	0.021*	0.125	0.237	0.144	0.171
Arteriolar hyalinosis (0–3)	0.196	0.062	0.081	0.442	0.298	0.004**
Chronic vascular microangiopathic lesions (0–9)	0.104	0.323	0.176	0.094	0.303	0.003**
Tubular atrophy (0–3)	−0.155	0.141	0.003	0.98	0.270	0.009**
Interstitial fibrosis (0–3)	−0.099	0.349	0.025	0.814	0.245	0.019**
Chronic tubulointerstitial lesions (0–6)	−0.135	0.200	0.011	0.919	0.261	0.012**

	IgA deposit		IgM deposit		IgG deposit	
	*r*	*p*	*r*	*p*	*r*	*p*
Glomerular endothelial cell swelling and proliferation (0–3)	−0.163	0.120	0.013	0.901	0.040	0.704
Glomerular subendothelial edema on electron microscopy (0–3)	0.030	0.780	0.105	0.320	0.003	0.976
Glomerular mesangialysis (0–1)	−0.076	0.473	0.263	0.011*	−0.069	0.515
Glomerular thrombi/necrosis (0–2)	0.054	0.611	0.054	0.608	0.072	0.495
Acute glomerular microangiopathic lesions (0–9)	−0.049	0.643	0.116	0.270	0.022	0.835
Glomerular basement membrane double contour (0–3)	−0.147	0.162	0.187	0.074	0.080	0.449
Mesangial proliferation (0–3)	−0.083	0.429	−0.082	0.437	0.078	0.462
Ischemic wrinkle of glomeruli (0–3)	0.073	0.489	0.081	0.442	0.215	0.040*
Global and segmental sclerosis (0–3)	−0.213	0.042*	−0.232	0.026*	−0.158	0.133
Chronic glomerular microangiopathic lesions (0–12)	−0.249	0.017*	−0.088	0.402	0.075	0.476
Endothelial swelling and subintimal myxoid edema of artery/arteriole (0–3)	−0.057	0.590	0.010	0.924	−0.070	0.508
Arteriolar/artery necrosis/thrombi (0–2)	−0.126	0.233	0.048	0.652	0.011	0.919
Acute vascular microangiopathic lesions (0–5)	−0.093	0.377	0.018	0.863	−0.052	0.620
Arteriolar onion skin lesions (0–3)	−0.104	0.326	−0.130	0.218	0.109	0.300
Intimal fibrosis of artery (0–3)	0.034	0.750	0.005	0.965	0.081	0.441
Arteriolar hyalinosis (0–3)	−0.022	0.835	0.063	0.552	0.075	0.476
Chronic vascular microangiopathic lesions (0–9)	−0.046	0.661	−0.025	0.810	0.132	0.210
Tubular atrophy (0–3)	−0.141	0.179	−0.266	0.010*	0.117	0.266
Interstitial fibrosis (0–3)	−0.125	0.236	−0.266	0.010*	0.079	0.456
Chronic tubulointerstitial lesions (0–6)	−0.136	0.197	−0.270	0.009**	0.105	0.321

	C1q deposit		C3 deposit		FRA deposit	
	*r*	*p*	*r*	*p*	*r*	*p*
Glomerular endothelial cell swelling and proliferation (0–3)	0.122	0.246	0.217	0.038*	−0.099	0.349
Glomerular subendothelial edema on electron microscopy (0–3)	0.131	0.212	0.014	0.892	0.121	0.252
Glomerular mesangialysis (0–1)	−0.069	0.515	0.139	0.186	0.012	0.907
Glomerular thrombi/necrosis (0–2)	0.069	0.516	0.057	0.589	−0.018	0.868
Acute glomerular microangiopathic lesions (0–9)	0.195	0.062	0.169	0.108	0.017	0.872
Glomerular basement membrane double contour (0–3)	0.212	0.043*	0.263	0.011*	−0.136	0.196
Mesangial proliferation (0–3)	−0.059	0.573	−0.023	0.830	−0.241	0.021*
Ischemic wrinkle of glomeruli (0–3)	0.052	0.623	0.192	0.066	0.048	0.649
Global and segmental sclerosis (0–3)	−0.048	0.650	−0.185	0.078	0.054	0.609
Chronic glomerular microangiopathic lesions (0–12)	0.075	0.476	0.303	0.779	−0.087	0.409
Endothelial swelling and subintimal myxoid edema of artery/arteriole (0–3)	0.158	0.133	0.045	0.673	0.138	0.190
Arteriolar/artery necrosis/thrombi (0–2)	0.130	0.216	0.105	0.318	−0.080	0.451
Acute vascular microangiopathic lesions (0–5)	0.196	0.061	0.076	0.469	0.077	0.467
Arteriolar onion skin lesions (0–3)	0.240	0.021*	0.025	0.182	0.120	0.253
Intimal fibrosis of artery (0–3)	−0.075	0.476	−0.135	0.200	−0.095	0.366
Arteriolar hyalinosis (0–3)	−0.027	0.799	−0.112	0.288	0.038	0.720
Chronic vascular microangiopathic lesions (0–9)	0.054	0.609	−0.115	0.273	0.038	0.720
Tubular atrophy (0–3)	0.219	0.036*	0.009	0.923	−0.144	0.171
Interstitial fibrosis (0–3)	0.232	0.026*	−0.042	0.688	−0.120	0.256
Chronic tubulointerstitial lesions (0–6)	0.288	0.029**	−0.014	0.894	−0.135	0.201

Abbreviations: eGFR: Estimated glomerular filtration rate; FRA: Fibrinogen.

Acute glomerular microangiopathic lesions consisted of glomerular endothelial cell swelling and proliferation, glomerular subendothelial edema on electron microscopy, glomerular mesangialysis and Glomerular thrombi/necrosis; Chronic glomerular microangiopathic lesions consisted of glomerular basement membrane double contour, mesangial proliferation, ischemic wrinkle of glomeruli and global and segmental sclerosis; Acute vascular microangiopathic lesions consisted of endothelial swelling and subintimal myxoid edema of artery/arteriole and arteriolar/artery necrosis/thrombi; Chronic vascular microangiopathic lesions consisted of arteriolar onion skin lesions, intimal fibrosis of artery and arteriolar hyalinosis; Chronic tubulointerstitial lesions consisted of tubular atrophy and interstitial fibrosis.

Correlations among nonparametric variables were analyzed using Spearman’s test.

* *p* < 0.05; **p < 0.01; ***p < 0.001.

### Associations between enrolled pathological lesions and clinical features

The correlation analysis between the 15 enrolled pathological variables above and clinical characteristics at the time of renal biopsy is shown in Table 3.

In briefly, (1) Predominant glomerular lesions were associated with the levels of proteinuria and serum albumin, as well as the deposit of IgA, IgM, IgG, C3, and fibrinogen in glomeruli. (2) Predominant vascular lesions were associated with the levels of serum creatinine and eGFR. (3) Overlap lesions, including glomerular and vascular lesions, were associated with the levels of hemoglobin and platelet count, and the deposit of C1q in glomeruli.

### Clinicopathological comparisons between the dialysis group and nondialysis group

Our patients were further grouped by dialysis at baseline into a dialysis group (with dialysis at the time of renal biopsy) and a nondialysis group (without dialysis at the time of renal biopsy).

Their clinical and pathological parameters are compared in Table 4.

**Table 4.** Clinicopathologic comparison between dialysis group and nondialysis group.

	Dialysis^a^	Nondialysis^a^	*p* ^b^
N (%)	46 (50.0)	46 (50.0)	
Clinical evaluation			
Sex (male/female), no.	26/20	33/13	0.13
Age (years), mean ± SD	34.46 ± 10.89	34.83 ± 8.11	0.854
Fever, no. (%)	6 (13.0)	2 (4.3)	0.141
Diabetes, no. (%)	0 (0)	3 (6.5)	0.08
Hematuria, no. (%)	27 (58.7)	13 (28.3)	0.003**
Liver involvement, no. (%)	5 (0.9)	3 (6.5)	0.462
Cardiac involvement, no. (%)	0 (0)	1 (2.2)	0.317
Neurologic disorder, no. (%)	5 (10.9)	3 (6.5)	0.462
Renal cortical necrosis, no. (%)	6 (13.0)	0 (0)	0.012*
Hematologic involvement, no. (%)	33 (71.7)	10 (21.7)	<0.001***
Musculoskeletal system involvement, no. (%)	1 (2.2)	0 (0)	0.317
Etiology, no. (%)			0.013*
aHUS	12 (26.1)	7 (15.2)	
P-HUS	14 (30.4)	6 (13.1)	
Malignant hypertension of unknown cause	20 (43.5)	33 (71.7)	
Laboratory Assessment			
Serum creatinine (mg/dL), median (range)	9.55 (4.14–33.03)	2.81 (0.62–7.59)	<0.001***
Urine protein (g/24h), median (range)	1.98 (0.15–14.31)	2.20 (0.03–7.57)	0.223
Albumin (g/L), mean ± SD	35.39 ± 6.17	38.72 ± 5.36	0.007**
Hemoglobin (g/dL), median (range)	69 (33–133)	129 (60–170)	<0.001***
Platelet count (×10^3^/mL), median (range)	86 (15–343)	200 (46–397)	<0.001***
eGFR (mL/min/1.73m^2^), median (range)	6.36 (1.50–13.60)	27.60 (7.80–115.10)	<0.001***
LDH (U/L), median (range), *n* = 69^c^	600 (139–7226)	206 (103–2143)	<0.001***
Serum C3 (g/L), mean ± SD, *n* = 85^d^	0.83 ± 0.25	1.00 ± 0.24	0.003**
Serum C4 (g/L), mean ± SD, *n* = 72^e^	0.23 ± 0.10	0.26 ± 0.09	0.191
Medications, no. (%)			
PE and/or PI	22 (47.8)	4 (8.7)	<0.001***
Steroids and other immunosuppressants	23 (50.0)	10 (21.7)	0.003**
Cyclophosphamide	6 (13.0)	4 (8.7)	0.505
RAAS blockades	32 (69.6)	36 (78.3)	0.345
Follow-up			
Duration of follow-up, month, median (range)	2 (1–13)	35 (3–209)	0.997
Dialysis at baseline, no. (%)	100 (100)	–	
Patients on dialysis at baseline who discontinued during the study period, no. (%)	26 (56.5)	–	
Nondialysis at baseline, no. (%)	–	100 (100)	
Composite events, no. (%)	–	19 (41.3)	
Death, no. (%)	–	1 (2.2)	
ESKD, no. (%)	–	19 (41.3)	
Renal Histopathology			
Glomerular endothelial cell swelling and proliferation (0–3)	0.63 ± 0.68	0.50 ± 0.66	0.317
Glomerular subendothelial edema on electron microscopy (0–3)	0.93 ± 0.85	0.70 ± 0.73	0.178
Glomerular mesangialysis (0–1)	0.04 ± 0.21	0.11 ± 0.31	0.241
Glomerular thrombi/necrosis (0–2)	0.69 ± 0.96	0.17 ± 0.57	0.505
Acute glomerular microangiopathic lesions (0–9)	2.30 ± 1.82	1.57 ± 1.83	0.098
Glomerular basement membrane double contour (0–3)	0.30 ± 0.55	0.28 ± 0.58	0.69
Mesangial proliferation (0–3)	0.37 ± 0.57	0.33 ± 0.56	0.669
Ischemic wrinkle of glomeruli (0–3)	2.09 ± 1.23	1.48 ± 1.11	0.012*
Global and segmental sclerosis (0–3)	1.63 ± 0.83	1.96 ± 0.87	0.071
Chronic glomerular microangiopathic lesions (0–12)	4.37 ± 1.62	4.04 ± 1.23	0.176
Endothelial swelling and subintimal myxoid edema of artery/arteriole (0–3)	2.24 ± 0.99	1.57 ± 1.09	0.003**
Arteriolar/artery necrosis/thrombi, (0–2)	0.74 ± 1.32	0.52 ± 0.22	0.016*
Acute vascular microangiopathic lesions (0–5)	2.80 ± 1.59	1.65 ± 1.78	0.001**
Arteriolar onion skin lesions (0–3)	1.98 ± 1.24	1.67 ± 1.19	0.199
Intimal fibrosis of artery (0–3)	1.42 ± 1.45	2.00 ± 1.19	0.095
Arteriolar hyalinosis (0–3)	0.74 ± 0.49	0.54 ± 0.94	0.023*
Chronic vascular microangiopathic lesions (0–9)	3.54 ± 1.93	4.42 ± 1.84	0.205
Tubular atrophy (0–3)	1.96 ± 0.99	1.98 ± 0.88	0.957
Interstitial fibrosis (0–3)	1.87 ± 1.00	1.96 ± 0.87	0.66
Chronic tubulointerstitial lesions (0–6)	3.83 ± 1.97	3.93 ± 1.74	0.831
Renal immunofluorescence			
IgA, median (range)	0 (0–2)	0 (0–2)	0.097
IgM, median (range)	0 (0–3)	0 (0–3)	0.612
IgG, median (range)	0 (0–2)	0 (0–2)	0.428
C1q, median (range)	0 (0–1)	0 (0–1)	1
C3, median (range)	0 (0–3)	0 (0–3)	0.673
ALB, median (range)	0 (0–1)	0 (0–1)	0.562
FRA, median (range)	0 (0–2)	0 (0–2)	0.124

Abbreviations: aHUS: Atypical hemolytic uremic syndrome; P-HUS: Pregnancy- and postpartum-associated hemolytic uremic syndrome; eGFR: Estimated glomerular filtration rate; LDH: Lactate dehydrogenase; PE: Plasma exchange; PI: Plasma infusion; RAAS: Rennin–angiotensin–aldosterone system; ESKD: End-stage kidney disease; ALB: Albumin; FRA: Fibrinogen.

* *p* < 0.05; **p < 0.01; ***p < 0.001.

^a^ Patients were grouped by dialysis use at baseline.

^b^ Between dialysis group and nondialysis group.

^c^ LDH detected among 69 patients, dialysis group (*n* = 41), nondialysis group (*n* = 28).

^d^ Serum C3 detected among 85 patients, dialysis group (*n* = 44), nondialysis group (*n* = 41).

^e^ Serum C4 detected among 72 patients, dialysis group (*n* = 36), nondialysis group (*n* = 36).

### Pathological lesions and prognosis of C-TMA patients

**Figure 3.**
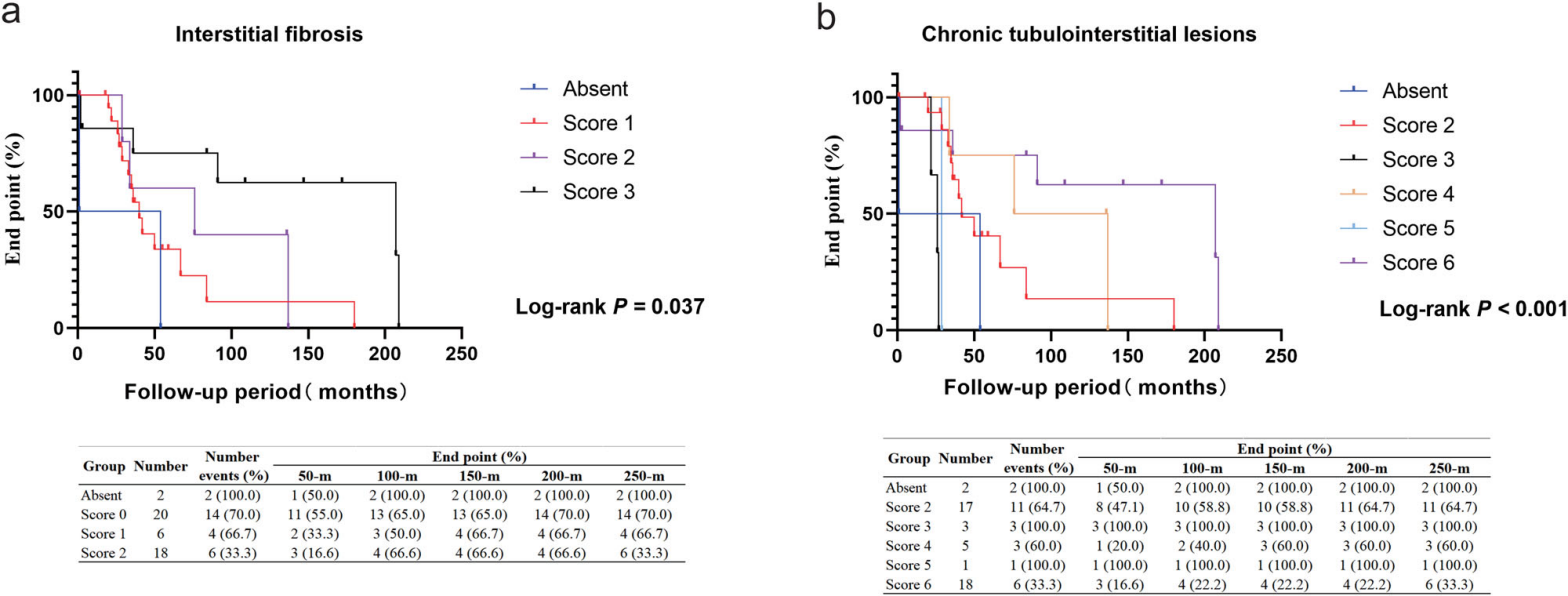
Kaplan–Meier analysis of different pathological features in dialysis group. (a) Kaplan–Meier analysis of discontinuing dialysis between the absent, score 1, score 2, and score 3 of interstitial fibrosis. (b) Kaplan–Meier analysis of discontinuing dialysis between the absent, score 2, score 3, score 4, score 5, and score 6 of chronic tubulointerstitial lesions.

Among the dialysis group, the univariate Cox regression analysis showed that severe interstitial fibrosis (HR, 95% CI, 0.124 (0.021–0.729), *p* = 0.021) and chronic tubulointerstitial lesions (HR, 95% CI, 0.116 (0.019–0.692), *p* = 0.018) were related to a trend of higher risk of continuous dialysis, details were shown in Supplementary Table S4, also presented by the Kaplan–Meier curve (Figure 3). The multivariable Cox hazard analysis further identified similar results after adjustment for sex, age, eGFR with or without proteinuria and hematuria (Table 5).

**Table 5.** Correlations between pathological features and discontinuing dialysis outcomes in dialysis group of 46 patients.

	Univariate		Model^a^		Model^b^	
	HR (95% CI)	*p*	HR (95% CI)	*p*	HR (95% CI)	*p*
Interstitial fibrosis						
Absent	1	Reference	1	Reference	1	Reference
Score 1	0.519 (0.116 − 2.334)	0.393	0.519 (0.116 − 2.334)	0.393	0.387 (0.081 − 1.859)	0.236
Score 2	0.334 (0.058 − 1.913)	0.218	0.334 (0.058 − 1.913)	0.218	0.135 (0.020 − 0.926)	0.041
Score 3	0.124 (0.021 − 0.729)	0.021	0.124 (0.021 − 0.729)	0.021	0.035 (0.004 − 0.228)	0.002
Chronic tubulointerstitial lesions						
Absent	1	Reference	1	Reference	1	Reference
Score 2	0.432 (0.093 − 2.000)	0.283	0.307 (0.062 − 1.519)	0.148	0.294 (0.059 − 1.457)	0.134
Score 3	3.612 (0.466 − 28.015)	0.219	5.572 (0.699 − 44.428)	0.105	2.224 (0.268 − 18.480)	0.460
Score 4	0.249 (0.039 − 1.597)	0.143	0.202 (0.030 − 1.349)	0.099	0.147 (0.021 − 1.037)	0.054
Score 5	1.871 (0.150 − 23.475)	0.626	1.494 (0.118 − 18.866)	0.756	0.728 (0.050 − 10.541)	0.816
Score 6	0.116 (0.019 − 0.692)	0.018	0.091 (0.014 − 0.580)	0.011	0.048 (0.006 − 0.370)	0.004

Univariate Cox regression analysis was used between all pathological changes and prognosis.

Abbreviations: HR: Hazard ratio; 95% CI: 95% confidence interval; eGFR: Estimated glomerular filtration rate.

Model^a^ was adjusted for age, sex, eGFR.

Model^b^ was adjusted for age, sex, eGFR, proteinuria, and hematuria (absent or present).

**Figure 4.**
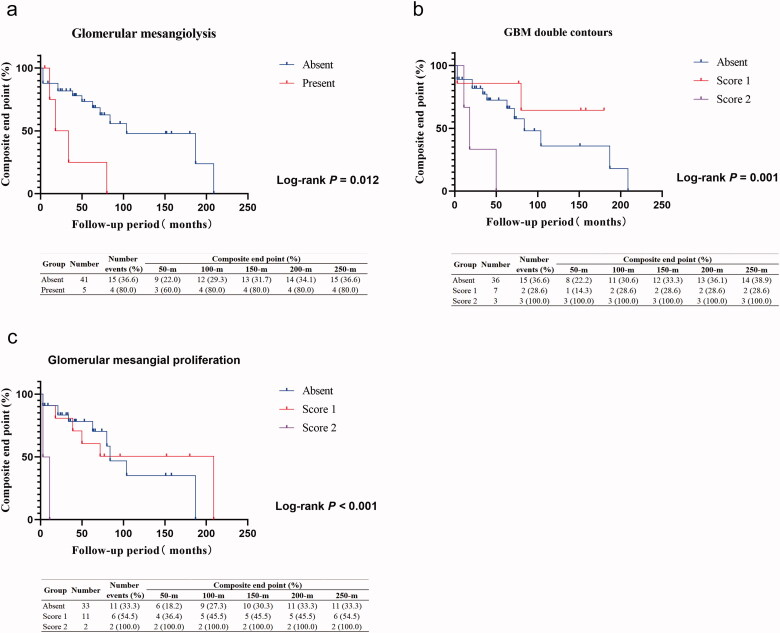
Kaplan–Meier analysis of different pathological features in nondialysis group. (a) Kaplan–Meier analysis of composite endpoints between the absent and present glomerular mesangiolysis group. (b) Kaplan–Meier analysis of composite endpoints among the group absent, score 1, and score 2 of glomerular basement membrane (GBM) double contours. (c) Kaplan–Meier analysis of composite endpoints among the group absent, score 1, and score 2 of glomerular mesangial proliferation.

Among the nondialysis group, the univariate Cox regression analysis showed that glomerular mesangiolysis (HR, 95% CI, 3.693 (1.160–11.753), *p* = 0.027), GBM double contours (HR, 95% CI, 4.830 (1.281–18.216), *p* = 0.020) and glomerular mesangial proliferation (HR, 95% CI, 14.660 (2.607–82.446), *p* = 0.002) were risk factors for worse compound kidney prognosis, also presented by the Kaplan–Meier curve (Figure 4). The multivariable Cox hazard analysis further identified similar results after adjustment for sex, age, eGFR with or without proteinuria and hematuria (Table 6).

**Table 6.** Summary of Cox regression models for the composite end points in nondialysis group of 46 patients.

	Univariate		Model^a^		Model^b^	
	HR (95% CI)	*p*	HR (95% CI)	*p*	HR (95% CI)	*p*
Glomerular mesangiolysis						
Absent	1	Reference	1	Reference	1	Reference
Score 1	3.693 (1.160 − 11.753)	0.027	3.693 (1.160 − 11.753)	0.027	3.693 (1.160 − 11.753)	0.027
Glomerular basement membrane double contour						
Absent	1	Reference	1	Reference	1	Reference
Score 1	0.486 (0.106 − 2.231)	0.353	0.240 (0.048 − 1.199)	0.082	0.292 (0.055 − 1.538)	0.146
Score 2	4.830 (1.281 − 18.216)	0.020	8.322 (1.990 − 34.803)	0.004	17.118 (3.503 − 83.640)	<0.001
Glomerular mesangial proliferation						
Absent	1	Reference	1	Reference	1	Reference
Score 1	0.854 (0.292 − 2.495)	0.773	0.987 (0.329 − 2.965)	0.982	1.279 (0.405 − 4.041)	0.675
Score 2	14.660 (2.607 − 82.446)	0.002	23.856 (3.737 − 152.298)	0.001	10.981 (1.435 − 84.028)	0.021

Univariate Cox regression analysis was used between all pathological changes and prognosis.

Abbreviations: HR: Hazard ratio; 95% CI: 95% confidence interval; eGFR: Estimated glomerular filtration rate.

Model^a^ was adjusted for age, sex, eGFR.

Model^b^ was adjusted for age, sex, eGFR, proteinuria, and hematuria (absent or present).

More importantly, as diagnostic tests and supportive measures etc. might differ between periods, our patients were divided into two groups, as group 1 (2000–2009) and group 2 (2010–2017), based on the period for further prognosis analysis.

In the dialysis subgroup and nondialysis subgroup of the ‘2000–2009’ cohort, the univariate Cox regression analysis did not reveal any risk pathological change for prognosis.

In the dialysis subgroup of the ‘2010–2017’ cohort, the univariate Cox regression analysis showed that severe chronic tubulointerstitial lesion (HR, 95% CI, 0.180 (0.010–0.903), *p* = 0.040) was related to a trend of a higher risk of continuous dialysis. The multivariable Cox hazard analysis further identified that severe chronic tubulointerstitial lesion (HR, 95% CI, 0.180 (0.010–0.903), *p* = 0.040) was related to a trend of a higher risk of continuous dialysis after adjustment for sex, age, eGFR with or without proteinuria and hematuria.

In the nondialysis subgroup of the ‘2010–2017’ cohort, the univariate Cox regression analysis showed that GBM double contours (HR, 95% CI, 5.470 (1.322–22.463), *p* = 0.018) was a risk factor for worse prognosis. The multivariable Cox hazard analysis further identified that GBM double contours (HR, 95% CI, 5.470 (1.322–22.463), *p* = 0.018) was a risk factor for worse prognosis after adjustment for sex, age, eGFR with or without proteinuria and hematuria.

## Discussion

C-TMA usually results from defective regulation of the alternative complement pathway, including atypical HUS, pregnancy- and postpartum-associated HUS, and malignant hypertension of unknown cause, which were enrolled in our cohort [4,5].

Recently, Kim summarized the histopathologic changes of TMA and emphasized the importance of a renal biopsy for TMA patients [25]. Habib et al. previously classified HUS renal pathology into glomerular, arterial, and cortical necrosis [26]. Although arterial lesions[15,27], arterial intimal thickening [28], arteriolar/arterial thrombosis, and thickening of the arterial medial and intimal layers [29,30] were shown to be associated with poor renal outcome in some studies, these studies included small sample sizes with evaluation of reproducibility. Thus, we attempted to establish a scoring system and tried to find specific TMA-related pathological features predicting clinical outcomes based on a well-defined C-TMA cohort. Herein, we extended the pathology dataset, including a total of 15 histopathologic variables, in our scoring system, and the majority of the 15 pathologic indices showed good or very good interobserver and intraobserver reproducibility, except glomerular mesangial proliferation, which showed moderate reliability. All 15 pathological changes were strongly associated with several clinical indices, including serum creatinine value, proteinuria, serum albumin, hemoglobin, and platelet count. We further found that patients with dialysis at onset had more severe acute vascular microangiopathic lesions, such as vascular endothelial cell swelling and subintimal myxoid edema and arteriolar/arterial fibrinoid necrosis/thrombi, than those without dialysis, which was similar to previous studies [28,29]. Morel-Maroger et al. showed that vascular lesions had a greater prognostic importance in acute renal failure with HUS among adult patients [28]. Mehrazma et al. also indicated that the probability of kidney disease among the patients with a vascular score of more than 0.14 was 5 times higher than those with a lower score in children with atypical HUS [29]. It should also be noticed that some of the correlations showed were not consistent with clinical practice or previous evidence, i.e., interstitial fibrosis negatively correlates with sCr etc. This could be due to several factors including the fact that acute/active lesions were more relevant than chronic changes when the biopsy was done etc.

The following information was found by our prognostic analysis. First, the initial dialysis patients with extensive interstitial fibrosis had a poor outcome by Cox hazard analysis, consistent with a previous study [26,31]. Second, the univariate analysis and Cox hazard multivariate analysis demonstrated that glomerular lesions, including glomerular mesangiolysis, GBM double counts, and glomerular mesangial proliferation, were risk factors for prognosis among patients without initial dialysis. These results all suggest that the current renal pathological scoring system is well correlated with prognosis in C-TMA patients. They also highlight the ‘cross-talk’ between pathophysiological phenotypes and precision treatment, especially targeted complement-specific bioagents. Interestingly, when we divided our patients into 2 groups, as 2000–2009 year group and 2010–2017 year group, based on the period for prognosis analysis, we found that the risk factor differed between the 2 groups. It might be due to the changes of diagnostic tests and supportive measures etc. as time goes by.

There are some limitations in our study. First, this was a single-center study. Second, as eculizumab was not approved by the China Food and Drug Administration (CFDA) before 2017, and no patient in our cohort received it. Further validation study with a ‘eculizumab’ cohort is needed.

In summary, our renal C-TMA pathological semiquantitative scoring system was reliable with good reproducibility and suggested to disease severity and clinical outcomes, which should be further validated with a larger dataset.

## Supplementary Material

Supplemental Material

## Data Availability

The datasets generated and/or analyzed during the current study are available from the corresponding author upon reasonable request.

## References

[1] Moake JL. Thrombotic microangiopathies. N Engl J Med. 2002;347(8):589–600. 12192020 10.1056/NEJMra020528

[2] George JN, Nester CM. Syndromes of thrombotic microangiopathy. N Engl J Med. 2014;371(7):654–666. 25119611 10.1056/NEJMra1312353

[3] Loirat C, Fakhouri F, Ariceta G, et al.; HUS International. An international consensus approach to the management of atypical hemolytic uremic syndrome in children. Pediatr Nephrol. 2016;31(1):15–39. 25859752 10.1007/s00467-015-3076-8

[4] Fakhouri F, Frémeaux-Bacchi V. Thrombotic microangiopathy in aHUS and beyond: clinical clues from complement genetics. Nat Rev Nephrol. 2021;17(8):543–553. 33953366 10.1038/s41581-021-00424-4

[5] Palomo M, Blasco M, Molina P, et al. Complement activation and thrombotic microangiopathies. Clin J Am Soc Nephrol. 2019;14(12):1719–1732. 31694864 10.2215/CJN.05830519 PMC6895490

[6] Gavriilaki E, Anagnostopoulos A, Mastellos DC. Complement in thrombotic microangiopathies: unraveling Ariadne’s thread into the labyrinth of complement therapeutics. Front Immunol. 2019;10:337. 30891033 10.3389/fimmu.2019.00337 PMC6413705

[7] Scully M, Cataland S, Coppo P, et al.; International Working Group for Thrombotic Thrombocytopenic Purpura. Consensus on the standardization of terminology in thrombotic thrombocytopenic purpura and related thrombotic microangiopathies. J Thromb Haemost. 2017;15(2):312–322. 27868334 10.1111/jth.13571

[8] Bayer G, von Tokarski F, Thoreau B, et al. Etiology and outcomes of thrombotic microangiopathies. Clin J Am Soc Nephrol. 2019;14(4):557–566. 30862697 10.2215/CJN.11470918 PMC6450353

[9] Fakhouri F, Scully M, Provôt F, et al. Management of thrombotic microangiopathy in pregnancy and postpartum: report from an international working group. Blood. 2020;136(19):2103–2117. 32808006 10.1182/blood.2020005221

[10] Brocklebank V, Wood KM, Kavanagh D. Thrombotic microangiopathy and the kidney. Clin J Am Soc Nephrol. 2018;13(2):300–317. 29042465 10.2215/CJN.00620117 PMC5967417

[11] Manenti L, Gnappi E, Vaglio A, et al. Atypical haemolytic uraemic syndrome with underlying glomerulopathies. A case series and a review of the literature. Nephrol Dial Transplant. 2013;28(9):2246–2259. 23787552 10.1093/ndt/gft220

[12] Lusco MA, Fogo AB, Najafian B, et al. AJKD atlas of renal pathology: thrombotic microangiopathy. Am J Kidney Dis. 2016;68(6):e33–e34. 27884283 10.1053/j.ajkd.2016.10.006

[13] Fakhouri F, Delmas Y, Provot F, et al. Insights from the use in clinical practice of eculizumab in adult patients with atypical hemolytic uremic syndrome affecting the native kidneys: an analysis of 19 cases. Am J Kidney Dis. 2014;63(1):40–48. 24021908 10.1053/j.ajkd.2013.07.011

[14] Hu Y-F, Tan Y, Yu X-J, et al. Podocyte involvement in renal thrombotic microangiopathy: a clinicopathological study. Am J Nephrol. 2020;51(9):752–760. 32862175 10.1159/000510141

[15] Yu X-J, Yu F, Song D, et al. Clinical and renal biopsy findings predicting outcome in renal thrombotic microangiopathy: a large cohort study from a single institute in China. ScientificWorldJournal. 2014;2014:680502. 2014:25184151 10.1155/2014/680502 PMC4144312

[16] Goodship THJ, Cook HT, Fakhouri F, et al.; Conference Participants. Atypical hemolytic uremic syndrome and C3 glomerulopathy: conclusions from a “kidney disease: improving global outcomes” (KDIGO) controversies conference. Kidney Int. 2017;91(3):539–551. 27989322 10.1016/j.kint.2016.10.005

[17] Levey AS, Stevens LA, Schmid CH, et al.; CKD-EPI (Chronic Kidney Disease Epidemiology Collaboration). A new equation to estimate glomerular filtration rate. Ann Intern Med. 2009;150(9):604–612. 19414839 10.7326/0003-4819-150-9-200905050-00006 PMC2763564

[18] Fakhouri F, Hourmant M, Campistol JM, et al. Terminal complement inhibitor eculizumab in adult patients with atypical hemolytic uremic syndrome: a single-arm, open-label trial. Am J Kidney Dis. 2016;68(1):84–93. 27012908 10.1053/j.ajkd.2015.12.034

[19] Caprioli J, Noris M, Brioschi S, et al.; International Registry of Recurrent and Familial HUS/TTP. Genetics of HUS: the impact of MCP, CFH, and IF mutations on clinical presentation, response to treatment, and outcome. Blood. 2006;108(4):1267–1279. 16621965 10.1182/blood-2005-10-007252 PMC1895874

[20] Fogo AB, Kashgarian M. Chapter 8 – renal transplantation. In: Fogo A.B, Kashgarian M, editors. Diagnostic atlas of renal pathology. 3rd ed. Amsterdam, Netherlands: Elsevier; 2017. p. 463–489.

[21] Armstrong GD. The intraclass correlation as a measure of interrater reliability of subjective judgments. Nurs Res. 1981;30(5):314–315, 320a. 6912993

[22] Landis JR, Koch GG. The measurement of observer agreement for categorical data. Biometrics. 1977;33(1):159–174. 843571

[23] Koch GG, Landis JR, Freeman JL, et al. A general methodology for the analysis of experiments with repeated measurement of categorical data. Biometrics. 1977;33(1):133–158. 843570

[24] Barbour SJ, Coppo R, Zhang H, et al.; International IgA Nephropathy Network. Evaluating a new international risk-prediction tool in IgA nephropathy. JAMA Intern Med. 2019;179(7):942–952. 30980653 10.1001/jamainternmed.2019.0600 PMC6583088

[25] Kim YJ. A new pathological perspective on thrombotic microangiopathy. Kidney Res Clin Pract. 2022;41(5):524–532. 35791743 10.23876/j.krcp.22.010 PMC9576460

[26] Habib R, Gagnadoux MF, Broyer M. [Hemolytic-uremic syndrome in children and arterial hypertension]. Arch Mal Coeur Vaiss. 1981;74 Spec No:37–43. 6794528

[27] Taylor CM, Chua C, Howie AJ, et al.; British Association for Paediatric Nephrology. Clinico-pathological findings in diarrhoea-negative haemolytic uraemic syndrome. Pediatr Nephrol. 2004;19(4):419–425. 14986082 10.1007/s00467-003-1385-9

[28] Morel-Maroger L, Kanfer A, Solez K, et al. Prognostic importance of vascular lesions in acute renal failure with microangiopathic hemolytic anemia (hemolytic-uremic syndrome): clinicopathologic study in 20 adults. Kidney Int. 1979;15(5):548–558. 480787 10.1038/ki.1979.70

[29] Mehrazma M, Hooman N, Otukesh H. Prognostic value of renal pathological findings in children with atypical hemolytic uremic syndrome. Iran J Kidney Dis. 2011;5(6):380–385. 22057069

[30] Batal I, Domsic RT, Shafer A, et al. Renal biopsy findings predicting outcome in scleroderma renal crisis. Hum Pathol. 2009;40(3):332–340. 18973923 10.1016/j.humpath.2008.08.001

[31] Thoenes W, John HD. Endotheliotropic (hemolytic) nephroangiopathy and its various manifestation forms (thrombotic microangiopathy, primary malignant nephrosclerosis, hemolytic-uremic syndrome). Klin Wochenschr. 1980;58(4):173–184. 7382329 10.1007/BF01476776

