# Supplementary material for: Clinical value of the renal pathologic scoring system in complement-mediated thrombotic microangiopathy
Source: Ren Fail. 2023 Jan 17;45(1):2161396. doi: 10.1080/0886022X.2022.2161396 (PMC9848373; doi:10.1080/0886022X.2022.2161396)
Supplement: Supplemental Material [file IRNF_A_2161396_SM2650.pdf]

Table S1. Score criteria sheet.

(A) Score sheet of glomerular microangiopathic lesions in renal C-TMA.

Glomerular microangiopathic lesion: 0-21	Criteria	Score
Acute glomerular microangiopathic lesions: 0-9		
Glomerular endothelial cell swelling and proliferation: 0-3	Absent remarkable change in endothelial cells.	0
	Focal (involving <50% glomeruli) and segmental (involving less than half of the glomerular tuft) swelling and proliferation of endothelial cells.	1
	Diffusely (involving ⩾50% glomeruli) moderate proliferation of endothelial cells.	2
	Diffusely (involving ⩾50% glomeruli) severe proliferation of endothelial cells with obliteration of capillary lumens.	3
Glomerular subendothelial edema on electron microscopy: 0-3	Absent subendothelial widening.	0
	Segmental mild widening of subendothelial zone.	1
	Diffusely moderate widening of subendothelial zone.	2
	Severely widening of subendothelial zone, with narrowed capillary lumens of ⩾50% in diameter.	3
Glomerular mesangialysis: 0-1	Absent	0
	⩾ 1 mesangial area	1
Glomerular necrosis/thrombi: (0-1) * 2	Absent	0
	⩾ 1 necrosis lesion or 1 thrombi	2

Information Classification: General

Table S1. Continued

	Criteria	Score
Global and segmental glomerulosclerosis (Including ischemic sclerotic glomeruli): 0-3	Absent segmental and global glomerular sclerosis.	0
	Less than 25% of glomeruli with segmental and global sclerosis.	1
	25%-50% of glomeruli with segmental and global sclerosis.	2
	More than 50% of glomeruli with segmental and global sclerosis.	3

(B) Score sheet of vascular microangiopathic lesions in renal C-TMA.

Vascular microangiopathic lesions: 0-14	Criteria	Score
Acute vascular microangiopathic lesions: 0-5		
Vascular endothelial cell swelling and subintimal myxoid edema: 0-3	Absent swelling of endothelial cells, and Absent intimal edematous change in arteriolar and arterial walls.	0
	Mild intimal edematous expansion, with up to 25% narrowing of lumen in affected arterioles; or endothelial cells swelling and proliferation without intimal edematous expansion.	1
	Moderate to severe intimal edematous expansion with 25%-50% narrowing of lumens in most affected arterioles.	2
	Severe intimal edematous expansion with more than 50% narrowing of lumens of arterioles.	3
Arteriolar/artery fibrinoid necrosis/thrombi: (0-1) * 2	Absent	0
	⩾ 1 necrosis lesion or 1 thrombi	2

Information Classification: General

Table S1. Continued

Chronic vascular microangiopathic lesions: 0-9		
Onion skin lesions: 0-3	Absent onion skin change.	0
	Less than 25% artery/arteriole with onion skin lesion.	1
	25%~50% artery/arteriole with onion skin lesion.	2
	More than 25% artery/arteriole with onion skin lesion.	3
Intimal fibrosis: 0-3	Absent intimal fibrosis in arteries and arterioles.	0
	Fibrointimal thickening with up to 25% of luminal narrowing of vessels in most affected vessels.	1
	25%-50% narrowing of lumens in most affected vessels.	2
	More than 50% narrowing of lumens in most affected vessels.	3
Arteriolar hyalinosis: 0-3	Absent PAS positive hyaline in arterioles.	0
	Less than 25% of arteriolar walls occupied by hyaline in most affected vessels.	1
	25%-50% of arteriolar walls occupied by hyaline in most affected vessels.	2
	More than 50% of arteriolar walls occupied by hyaline in most affected vessels.	3

Information Classification: General

Table S1. Continued

(C) Score sheet of chronic tubulointerstitial lesions in renal C-TMA.

Vascular microangiopathic lesions: 0-6	Criteria	Score
Tubular atrophy: 0-3	Absent tubular atrophy.	0
	Mild tubular atrophy involved less than 25% of cortex.	1
	Moderate tubular atrophy involved 25%-50% of cortex.	2
	Severe tubular atrophy involved more than 50% of cortex.	3
Interstitial fibrosis: 0-3	Absent interstitial fibrosis.	0
	Mild interstitial fibrosis involved less than 25% of cortex.	1
	Moderate interstitial fibrosis involved 25%-50% of cortex.	2
	Severe interstitial fibrosis involved more than 50% of cortex.	3

Information Classification: General

Table S2. Distribution of the histopathologic findings.

Parameters	Scores	n=92	Parameters	Scores	n=92
Glomerular endothelial cell swelling and proliferation (0-3), n (%)	Score 0	49 (53.3)	Endothelial swelling and subintimal myxoid edema of artery/arteriole (0-3), n (%)	Score 0	9 (9.8)
	Score 1	35 (38.0)		Score 1	32 (34.8)
	Score 2	8 (8.7)		Score 2	10 (10.9)
	Score 3	0 (0)		Score 3	41 (44.6)
Glomerular subendothelial edema on electron microscopy (0-3), n (%)	Score 0	36 (39.1)	Arteriolar/artery necrosis/thrombi, (0-2), n (%)	Score 0	76 (82.6)
	Score 1	40 (43.5)			
	Score 2	13 (14.1)		Score 2	16 (17.4)
	Score 3	3 (3.3)			
Glomerular mesangialysis (0-1), n (%)	Score 0	85 (92.4)	Arteriolar onion skin lesions (0-3), n (%)	Score 0	19 (20.7)
	Score 1	7 (7.6)		Score 1	20 (21.7)
Glomerular thrombi/necrosis (0-2), n (%)	Score 0	82 (89.1)		Score 2	11 (12.0)
	Score 2	10 (10.9)		Score 3	42 (45.7)
Glomerular basement membrane double contour (0-3), n (%)	Score 0	70 (76.1)	Intimal fibrosis of artery (0-3), n (%)	Score 0	33 (35.9)
	Score 1	17 (18.5)		Score 1	3 (3.3)
	Score 2	5 (5.4)		Score 2	15 (16.3)
	Score 3	0 (0)		Score 3	41 (44.6)
Mesangial proliferation (0-3), n (%)	Score 0	62 (69.6)	Arteriolar hyalinosis (0-3), n (%)	Score 0	71 (71.2)
	Score 1	24 (26.1)		Score 1	13 (14.1)
	Score 2	4 (4.3)		Score 2	4 (4.3)
	Score 3	0 (0)		Score 3	4 (4.3)
Ischemic wrinkle of glomeruli (0-3), n (%)	Score 0	19 (20.7)	Tubular atrophy (0-3), n (%)	Score 0	3 (3.3)
	Score 1	21 (22.8)		Score 1	32 (34.8)
	Score 2	13 (14.1)		Score 2	22 (23.9)
	Score 3	39 (42.4)		Score 3	35 (38.0)
Global and segmental sclerosis (0-3), n (%)	Score 0	3 (3.3)	Interstitial fibrosis (0-3), n (%)	Score 0	3 (3.3)
	Score 1	36 (39.1)		Score 1	35 (38.0)
	Score 2	30 (32.6)		Score 2	21 (22.8)
	Score 3	23 (25.0)		Score 3	33 (35.9)

Information Classification: General

Table S3. Pathology dataset: intra-observer reproducibility.

Parameters	ICC
Glomerular endothelial cell swelling and proliferation	0.880***
Glomerular subendothelial edema on electron microscopy	0.949***
Glomerular mesangiolysis	1.000***
Glomerular necrosis/thrombi	1.000***
GBM double contours	0.910***
Glomerular mesangial proliferation	0.659***
Ischemic wrinkle of GBM	0.979***
Global and segmental glomerulosclerosis	0.956***
Vascular endothelial cell swelling and subintimal myxoid edema	0.967***
Arteriolar/artery fibrinoid necrosis/thrombi	0.788***
Arteriolar onion skin lesion	0.967***
Intimal fibrosis of artery	0.982***
Arteriolar hyalinosis	0.959***
Tubular atrophy	0.950***
Interstitial fibrosis	0.915***

***, P<0.001; GBM, glomerular basement membrane; ICC, intraclass correlation coefficient.

Information Classification: General

Table S4. univariate Cox regression analysis between all pathological changes and prognosis in dialysis group.

	Univariate	
	HR (95% CI)	p
Age	0.982 (0.935 - 1.032)	0.481
Sex	1.304 (0.579 - 2.936)	0.522
Serum creatinine	0.954 (0.884 - 1.029)	0.224
eGFR	1.093 (0.959 - 1.246)	0.181
Proteinuria	0.855 (0.177 - 0.878)	0.026
Hematuria	0.310 (0.111 - 0.864)	0.025
Complement C3	0.882 (0.199 - 3.914)	0.868
Glomerular endothelial cell swelling and proliferation		
Absent	1	Reference
Score 1	6.035 (0.746 - 34.839)	0.092
Score 2	5.016 (0.642 - 39.171)	0.124
Glomerular subendothelial edema on electron microscopy		
Absent	1	Reference
Score 1	0.490 (0.196 - 1.224)	0.127
Score 2	0.259 (0.136 - 1.553)	0.211
Score 3	1.307 (0.163 - 10.465)	0.801
Glomerular mesangialysis (0-1)		
Absent	1	Reference
Score 1	0.041 (0 - 20.072)	0.312
Glomerular thrombi/necrosis (0-2)		
Absent	1	Reference
Score 2	0.583 (0.232 - 1.464)	0.251
Acute glomerular microangiopathic lesions (0-9)		
Score 1	1	Reference
Score 2	0.755 (0.219 - 2.599)	0.656
Score 3	0.953 (0.263 - 3.453)	0.942
Score 4	0.575 (0.126 - 2.629)	0.275
Score 5	0.745 (0.166 - 3.344)	0.700
Score 6	7.240 (0.566 - 92.679)	0.128
Score 7	0.005 (0 - 158.313)	0.314
Score 8	0	0.996
Glomerular basement membrane double contour (0-3)		
Absent	1	Reference
Score 1	0.379 (0.126 - 1.147)	0.086
Score 2	0	0.984

Information Classification: General

Table S4. Continued

	Univariate	
	HR (95% CI)	p
Mesangial proliferation (0-3)		
Absent	1	Reference
Score 1	0.514 (0.202 - 1.306)	0.162
Score 2	0	0.989
Ischemic wrinkle of glomeruli (0-3)		
Absent	1	Reference
Score 1	1.650 (0.449 - 6.063)	0.451
Score 2	1.429 (0.399 - 5.114)	0.583
Score 3	0.507 (0.179 - 1.437)	0.201
Global and segmental sclerosis (0-3)		
Absent	1	Reference
Score 1	0.850 (0.108 - 6.660)	0.877
Score 2	1.080 (0.131 - 8.918)	0.843
Score 3	1.629 (0.178 - 14.931)	0.666
Chronic glomerular microangiopathic lesions (0-12)		
Score 1	1	Reference
Score 2	0.746 (0.122 - 4.575)	0.752
Score 3	1.471 (0.314 - 6.883)	0.624
Score 4	0.486 (0.118 - 1.998)	0.318
Score 5	0.666 (0.166 - 2.661)	0.565
Score 6	0.264 (0.043 - 1.656)	0.155
Score 7	0	0.978
Score 8	0	0.996
Endothelial swelling and subintimal myxoid edema of artery/arteriole (0-3)		
Absent	1	Reference
Score 1	0.786 (0.164 - 3.759)	0.763
Score 2	0.111 (0.010 - 1.277)	0.078
Score 3	0.355 (0.077 - 1.632)	0.183
Arteriolar/artery necrosis/thrombi (0-2)		
Absent	1	Reference
Score 2	0.610 (0.227 - 1.640)	0.327
Acute vascular microangiopathic lesions (0-5)		
Absent	1	Reference
Score 1	0.793 (0.165 - 3.796)	0.771
Score 2	0.109 (0.009 - 1.260)	0.076
Score 3	0.409 (0.085 - 1.963)	0.264
Score 5	0.279 (0.051 - 1.512)	0.139

Information Classification: General

Table S4. Continued

	Univariate	
	HR (95% CI)	p
Arteriolar onion skin lesions (0-3)		
Absent	1	Reference
Score 1	0.460 (0.140 - 1.509)	0.200
Score 2	0.608 (0.152 - 2.438)	0.483
Score 3	0.314 (0.113 - 1.073)	0.076
Intimal fibrosis of artery (0-3)		
Absent	1	Reference
Score 1	0	0.985
Score 2	0.713 (0.149 - 3.402)	0.671
Score 3	1.126 (0.473 - 2.679)	0.788
Arteriolar hyalinosis (0-3)		
Absent	1	Reference
Score 1	0.209 (0.027 - 1.588)	0.130
Score 2	2.688 (0.585 - 12.345)	0.204
Chronic vascular microangiopathic lesions (0-9)		
Absent	1	Reference
Score 1	0.573 (0.136 - 2.405)	0,447
Score 2	0.416 (0.074 - 2.323)	0.317
Score 3	0.292 (0.072 - 1.184)	0.085
Score 4	0.198 (0.036 - 1.098)	0.064
Score 5	0.498 (0.120 - 2.067)	0.337
Score 6	0.799 (0.197 - 3.239)	0.753
Score 7	0	0.996
Tubular atrophy (0-3)		
Absent	1	Reference
Score 1	0.435 (0.095 - 1.998)	0.285
Score 2	0.503 (0.098 - 2.590)	0.411
Score 3	0.162 (0.030 - 1.087)	0.066
Interstitial fibrosis (0-3)		
Absent	1	Reference
Score 1	0.519 (0.116 - 2.334)	0.393
Score 2	0.334 (0.058 - 1.913)	0.218
Score 3	0.124 (0.021 - 0.729)	0.021
Chronic tubulointerstitial lesions (0-6)		
Absent	1	Reference
Score 2	0.432 (0.093 - 2.000)	0.283
Score 3	3.612 (0.466 - 28.015)	0.219
Score 4	0.249 (0.039 - 1.597)	0.143
Score 5	1.871 (0.150 - 23.475)	0.626
Score 6	0.116 (0.019 - 0.692)	0.018

Univariate Cox regression analysis was used between all pathological changes and prognosis.

HR, hazard ratio; 95% CI, 95% confidence interval; eGFR, estimated glomerular filtration rate.

Model [a] was adjusted for age, sex, eGFR.

Model [b] was adjusted for age, sex, eGFR, proteinuria, and hematuria (absent or present).

Information Classification: General
